# Dynamic cardiac computed tomography characteristics of double-chambered right ventricle

**DOI:** 10.1038/s41598-022-25230-1

**Published:** 2022-11-29

**Authors:** Monal Yu-Hsuan Chang, Yan-De Liou, Jou-Hsuan Huang, Chia-Hung Su, Shu-Chien Huang, Ming-Tai Lin, Shyh-Jye Chen

**Affiliations:** 1 Department of Radiology and Medical Imaging, National Taiwan University Hospital, No. 7, Chung-Shan South Road, Taipei, 100 Taiwan; 2 Department of Surgery, National Taiwan University Hospital, No. 7, Chung-Shan South Road, 100 Taipei, Taiwan; 3 Department of Pediatrics, National Taiwan University Hospital, No. 7, Chung-Shan South Road, 100 Taipei, Taiwan

**Keywords:** Cardiovascular diseases, Paediatric research, Paediatrics, Medical imaging, Three-dimensional imaging, Tomography

## Abstract

To introduce image characteristics of double-chambered right ventricle on cardiac computed tomography and set a diagnostic criterion for the diagnosis. We retrospectively collected and measured the right ventricular constrictive ratio on computed tomography images in children who had simple ventricular septal defects in the past 10 years, because double-chambered right ventricle is often associated with ventricular septal defects. The right ventricular constrictive ratio was defined as the subinfundibular cross-sectional intraluminal area during end-systole divided by the area during end-diastole in the same patient. We compared the right ventricular constrictive ratio between subjects with concomitant double-chambered right ventricle and those without. 52 children were included, and 23 (44.2%) of them have concomitant double-chambered right ventricle. In most cases (*n *= 21; 91.3%), the hypertrophied muscular bundles occur just inferior to the level of the supraventricular crest in the right ventricle. Mean right ventricular constrictive ratio in patients with double-chambered right ventricle (15%) was significantly smaller than that without (29%). A cut-off value of a right ventricular constrictive ratio less than 20.1% was established to diagnose double-chambered right ventricle with an 89.7% sensitivity and 78. 3% specificity. Right ventricular constrictive ratio can be a valuable asset for the preoperative diagnosis of double-chambered right ventricle with cardiac computed tomography.

## Introduction

Double-chambered right ventricle (DCRV) is a rare congenital heart disease in which muscular bundles growing in the *subinfundibular* area are hypertrophied, separating the right ventricle (RV) into two chambers. The muscular bundles tend to cause right ventricular outflow tract obstruction (RVOTO). It is an entity not to be confused with the spectrum of diseases with pulmonary stenosis, such as Tetrology of Fallot, in which the infundibulum rather than the subinfundibulum is stenotic^1,2^. Almost 90% of DCRV cases have associated membranous ventricular septal defects (VSD)^3,4^. Some believed the hypertrophied muscular bundles in DCRV are induced by VSD, in order to prevent the pulmonary circulation from overloading^5^. A right intraventricular pressure gradient of at least 20 mmHg should be measured during transthoracic echocardiography for DCRV to be considered^2,3,6^. When treated properly with surgery, usually myectomy of the hypertrophied muscular bundles, the prognosis is optimal with complications occurring rarely^7–9^.

However, the subinfundibulum is not always well visualized in standard planes of transthoracic echocardiography, and measuring the RV pressure gradient is not a routine^10^. While most prospective ECG-gated cardiac computed tomography (CT) images disclose heart appearances during the diastolic phases, hypertrophied muscular bundles in DCRV are often reasonably more visible when the heart is contracted in systole^1,11,12^. Observation during surgery is also not always straightforward because operated hearts are usually in a state of induced diastolic arrest during cardiopulmonary bypass. All of which may lead to overlooking of underlying DCRV and thus reoperations when RVOTO gets worse^6,13,14^. Correct diagnosis before surgery is thereby crucial.

Numerous studies have already explored the diagnostic value of conventional echocardiography^1,3,15^ in DCRV. Data regarding its diagnosis via ECG-gated cardiac CT on the other hand is relatively scarce, while CT still remains a popular modality of choice in Asia^16^. We not only share our experience in diagnosing DCRV with cardiac CT in this study, to our knowledge this is also the first of its kind to gather such a considerable amount of cases and perform quantified measurements.

## Material and methods

### Patients

The National Taiwan University Hospital Research Ethics Committee has approved this retrospective study with survey, waiving the need for informed consent as it does not affect patient safety or clinical outcome. The study protocol strictly adhered to the ethical guidelines of the 1975 Declaration of Helsinki and was conducted in a single tertiary referral hospital.

We retrospectively collected information on patients diagnosed with simple VSD and received operations for this cause between March 2008 and October 2018. All patients received transthoracic echocardiography followed by retrospective ECG-gated cardiac CT within 3 months before surgery, as part of the routine in the surgical ward. Final confirmation of a DCRV diagnosis was made using intraoperative transesophageal echocardiography when RV pressure gradient reached 60 mmHg. Confirmed patients with DCRV form the study group, who would then receive subinfundibular myectomy along with VSD repair. The control group consisted of patients who did not have DCRV and only received VSD repair without myectomy.

Patients with complex heart diseases, such as Tetralogy of Fallot, transposition of the great arteries, double outlet right ventricle, endocardial cushion defect, hypoplastic left heart syndrome or heterotaxy were completely excluded from this study. Patients who received myectomy in the infundibulum instead of subinfundibulum were also not included in this study.

All participants underwent postoperative follow-up transthoracic echocardiography. No residual right ventricular pressure gradient or arrhythmia was noted in any of them in the control or study group.

### Cardiac CT acquisitions

A 64-detector scanner (Somatom Sensation 40; Siemens Medical, Erlangen, Germany) was used since December 2008, and another 320-detector scanner (Aquilion ONE; Canon Medical Systems, Japan) was used since December 2016. Data were acquired in the caudocranial direction, with a section thickness of 0.625–0.65 mm without gaps. Dynamic changes in the RV mid-cavity were explored using retrospectively gated cardiac CT. A cardiac cycle was divided into 20 phases, which means that each phase image was reconstructed at a 5% interval of the cardiac cycle. The matrix size in the X–Y plane was 512 × 512 pixels. A nonionic iodinated contrast medium (2 mL/kg Ultravist 370; Schering AG, Berlin, Germany) was delivered through a power injector to all patients at 80% of the maximum allowable injection rate. The delay time between the start of the contrast medium administration and the start of imaging was determined using a bolus tracking technique. A region in the ascending aorta was used for bolus-tracking; the threshold level was set at 150 HU. All cooperative patients were asked to hold their breath during scanning. Quiet respiration without crying was deemed acceptable in younger children and infants who could not comply with this request; therefore, patients aged < 5 years were routinely sedated with chloral hydrate (50 mg/kg) during examination. The mAs and kVp were age adjusted in order to reduce the radiation dose, based on the as low as reasonably achievable principle^17–19^.

### Post-processing techniques and quantifications

Image reconstruction and quantification were performed at an independent workstation using commercialized CT softwares (Syngo®; Siemens Medical Solutions, Forchheim, Germany / Vitrea® Advanced Visualization; Vital Images, Canon Medical Systems). Animated myocardial models with the viewer’s side (lateral anterior RV wall) removed were created for surgeons to better visualize the location of the muscular bundles if they exist (Supplemental video). The models we designed are slightly different from common 3D volume renderings in that instead of the contrast-filled intraluminal areas, we rendered the muscular portions instead, leaving chambers hollow.

A survey questionnaire was sent out to a group of physicians consisting of cardiologists and cardiovascular surgeons with examples of DCRV cases to clarify whether such reconstructed myocardial models are useful in the eyes of non-radiology physicians. They were asked to rate their confidence in pointing out the whereabouts of hypertrophied muscular bundles with three different image sets from the same DCRV patient on a scale of 1 to 5. The three image sets were a single static CT image, a dynamic cine CT loop, and an animated myocardial model.

**Figure 1:**
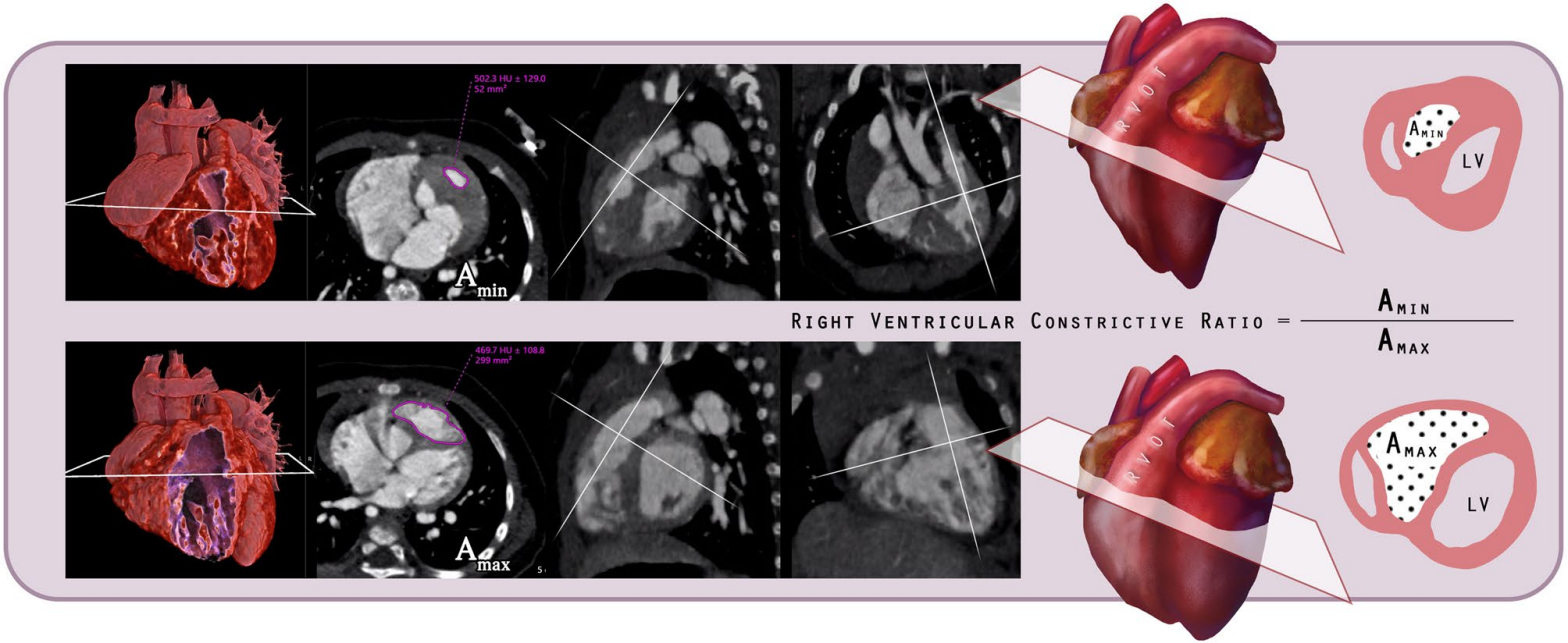
The patient was diagnosed with simple VSD and DCRV via CT. Surgeons performed a patch repair for VSD and resection of the hypertrophied muscular bundle. Three-dimensional myocardial model images of his heart during the end-systole (upper row) and end-diastole (lower row) are shown. The reformatted CT images from left to right are oblique axial, oblique sagittal, and oblique coronal sections in both upper and lower rows. Measured luminal areas A_min_ and A_max_ are outlined (in pink) on the cross-sectional images paralleled to the apparent muscular ridge. Right ventricular constrictive ratio (A_min_/A_max_) for this patient was 17.4%. [*CT *computed tomography; *DCRV* double-chambered right ventricle; *VSD *ventricular septal defect].

We localized the level of the supraventricular crest in RV during both end-systolic and end-diastolic phases. The cross-sectional images perpendicular to the central line of RVOT direction were obtained with multiplanar reformatting. The areas of the middle RV excluding large trabeculations on the cross-sectional images were measured. Areas measured in end-diastole and end-systole were defined as A_max_ and A_min_ respectively. The right ventricular constrictive ratio was defined as A_min_ divided by A_max_ (Fig. 1) in percentages. We also classified each DCRV patient into two types according to reported rules originally used for echocardiography^20^.

Two radiologists with 2 and 25 years of experience in congenital heart disease image interpretation assessed these images and obtained the required measurements. There was good interobserver agreement (overall intra-class correlation coefficient = 0.701) between interpreters with vast experience differences.

### Statistical analysis

Descriptive statistics and Student’s t test were used to compare the mean measurements of the study and control groups. The receiver operating characteristic curve analysis established a diagnostic CR cutoff value for DCRV. The preceding statistical analyses were performed using the commercially available software SPSS (version 25, Polar Engineering Inc., USA). A *p* value of < 0.05 was considered significant.

### Patient and public involvement

This is a retrospective pure image analysis study that did not involve patient or public recruitment prior to research and did not affect treatment decisions.

## Results

We enrolled 52 patients with simple VSD, in which 23 (44.2%) of them have DCRV (mean age: 1.1 years old; male: female = 14:9). The remaining 29 (55.8%) patients (mean age: 2 years old; male: female = 17:12) had simple VSD but no DCRV. Preoperative echocardiography diagnosed DCRV in 11 out of 23 patients (48%) with an average middle RV pressure gradient of 44.2 mmHg. (Table 1) Four patients received preoperative cardiac catheterization, three of them had it performed before coming to our institution and detailed datas were lost. The only one patient receiving preoperative cardiac catheterization at our hospital yielded an intraventricular pressure gradient of 50 mmHg.

**Table 1:** Patient demographics.

Characteristics	Study group (VSD + DCRV); *n* = 23 n (%)	Control group (VSD); *n* = 29 n (%)	*p* value
Age			0.209
Infants(< 1 year)	20 (87)	14 (48)	
Preschoolers(1–5 years)	2 (8)	12 (41)	
School-aged (> 5 years)	1 (5)	3 (11)	
Sex			0.951
Male	14 (61)	17 (59)	
**Preoperative DCRV diagnosis**			
Echocardiography	11 (48)	0 (0)	0.006
Mean right ventricular constrictive ratio	15%	29%	< 0.001

*CT* computed tomography; *DCRV* double-chambered right ventricle; *VSD* ventricular septal defect.

An extremely high degree of hypertrophied muscular bundles in our DCRV cases (*n* = 21; 91.3%) involve or occur just inferior to the level of supraventricular crest in the right ventricle.

**Figure 2:**
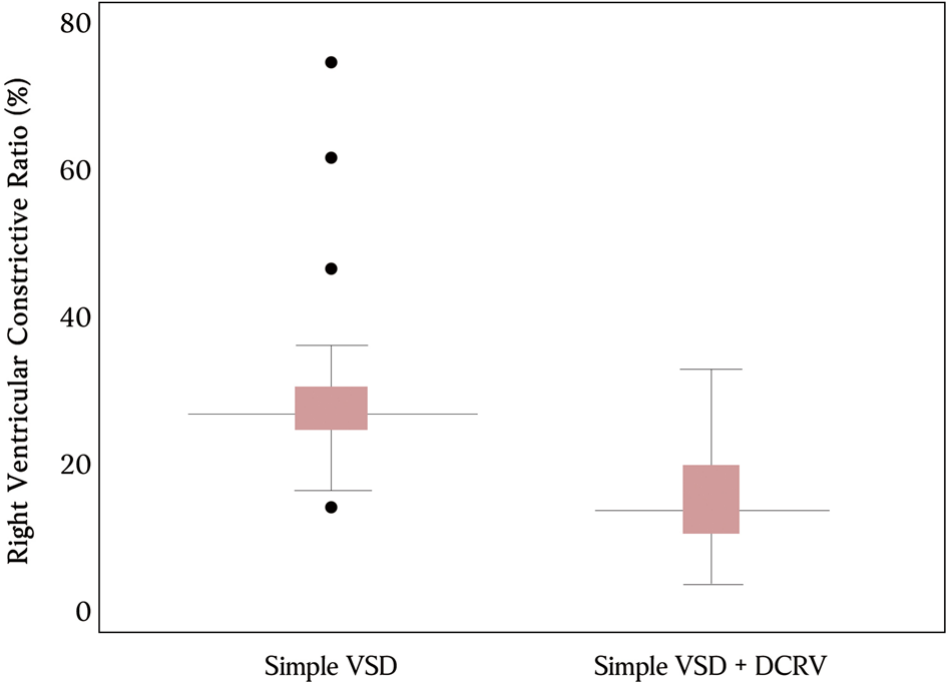
Boxplots showing data distribution of right ventricular constrictive ratio in percentages in simple ventricular septal defect (VSD) patients with or without double-chambered right ventricle (DCRV). Statistical significance was achieved with independent samples t-test (*p* < 0.001).

**Figure 3:**
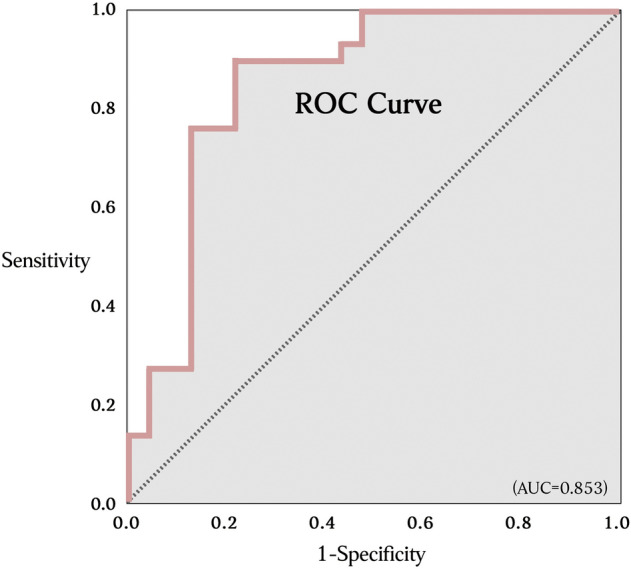
The high area under curve (AUC = 0.853) indicates good reliability of right ventricular constrictive ratio in double-chambered right ventricle prediction, with an optimal cutoff value of 20.1%. [*ROC *receiver operating characteristic].

Mean right ventricular constrictive ratio in patients with DCRV (15.0%) was significantly lower than that in patients without DCRV (29.0%), with *p* value < 0.001 (Fig. 2). There were a few outliers in the control group, which means that having a dramatic morphology does not necessarily translate to high RV pressure gradient, hence obstruction. Diagnosis accuracy can always be enhanced by correlating different imaging modalities with one another. A high area (0.853) under the receiver operating characteristic curve was indicative that right ventricular constrictive ratio is a reliable indicator for confirming DCRV (Fig. 3). A cut-off value of 20.1% was established as the diagnosis threshold. Patients with a right ventricular constrictive ratio of less than 20.1% were highly likely to have DCRV with an 89.7% sensitivity and 78.3% specificity.

According to our survey questionnaire collected from a group of 35 non-radiology pediatric cardiology specialists, the average confidence to point out the whereabouts of muscular bundles in DCRV on a scale of 1 to 5 (higher ratings for higher confidence) was 2.87 for single static CT image, 4.30 for dynamic cine CT loop, and 4.46 when myocardial models were present.

The average radiation effective dose of included patients was estimated to be 1.72 mSv in infants, 3.46 mSv in preschoolers, and 9.87 mSv in school-aged patients performed with our 64-detector CT scanner. Average radiation dosage was reduced to 0.43 mSv in infants and 1.61 mSv in preschoolers with our newly-installed 320-detector CT scanner. No school-aged patients received examinations with the 320-detector CT scanner. No sequelae from chloral hydrate sedation occurred in any of the patients.

### Comment

**Figure 4:**
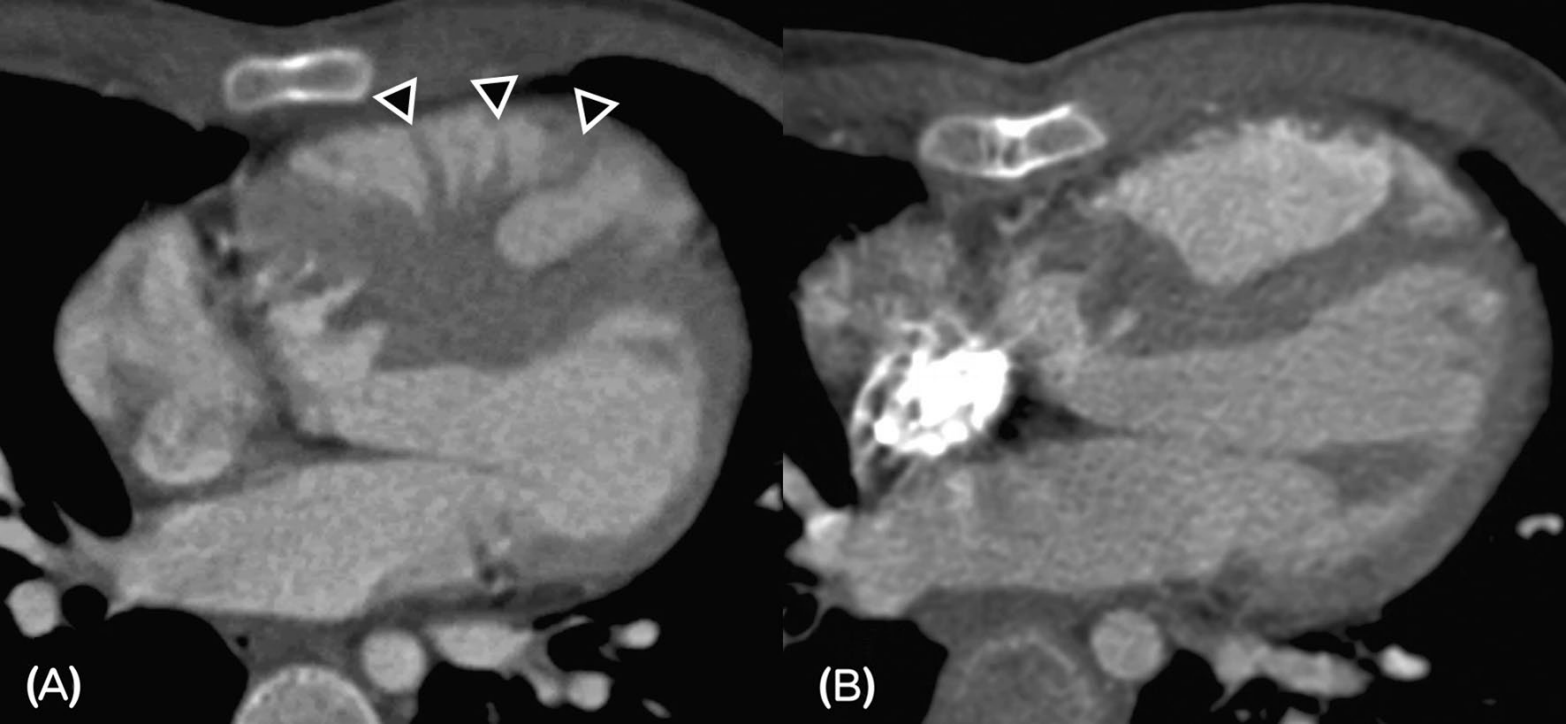
We provide a case of neglected DCRV as an example to emphasize the importance of excluding DCRV before a simple ventricular septal defect (VSD) repair. This patient received VSD repair at birth and had been leading a normal life until he was brought to our hospital due to progressive exertional dyspnea. Initial transthoracic echocardiography yielded increased pulmonary flow and an extremely high RV mid-cavity pressure gradient of over 125 mmHg. Preoperative dynamic cardiac CT raised suspicion for DCRV (right ventricular constrictive ratio = 3.6%) with identifiable muscle bundles crossing the RV cavity inferior to the level of the supraventricular crest with septal hypertrophy (**A**). The patient received reoperation to remove the muscle bundles and postoperative CT showed no more right ventricular outlet obstruction (**B**). The patient was completely free of symptoms after surgical resection of the hypertrophic muscles (arrows).

Surgical correction of DCRV often results in optimal outcomes with immediate relief of the RV pressure^6,21,22^. When simple VSD is repaired without simultaneously correcting DCRV, tension could build up in the patched-up ventricle as hypertrophied muscular bundles continue to cause mid-cavity obstruction, but now without a defect to partially release the ventricular pressure^9^. (Fig. 4) During surgery when the heart is flaccid, the job of spotting hypertrophied muscular bundles without prior notice or intraoperative transesophageal echocardiography may not be straightforward. Therefore, alerting surgeons of the possible presence of DCRV preoperatively becomes crucial.

Transthoracic echocardiography is considered the first-line screening examination for most cardiac diseases, including DCRV, although its accuracy may vary with different machines and operators^1,3,23^. When the subinfundibulum is poorly visualized, and the diagnosis is inconclusive, CT might step in as a reliable alternative. Retrospective ECG-gated cardiac CT provides an animated, non-invasive demonstration of DCRV with objective measurements of chamber size changes.

The heart is a moving organ ever-changing its forms and shapes during a single cardiac cycle. Hypertrophic muscular bundles in DCRV are more easily spotted when contracted and thickened during systolic phases^1,11^. (Supplementary video) While isolated DCRV is extremely rare, associated lesions are by contrast better demonstrated during diastolic phases^2,3,22^. That is the reason why both systolic and diastolic images are essential for DCRV cases. In intricate details, retrospective ECG-gated cardiac CT shows the entire cardiac cycle, both systole and diastole. Identifying perplexity in congenital heart diseases has always been a strong suit for CT^1^.

**Figure 5:**
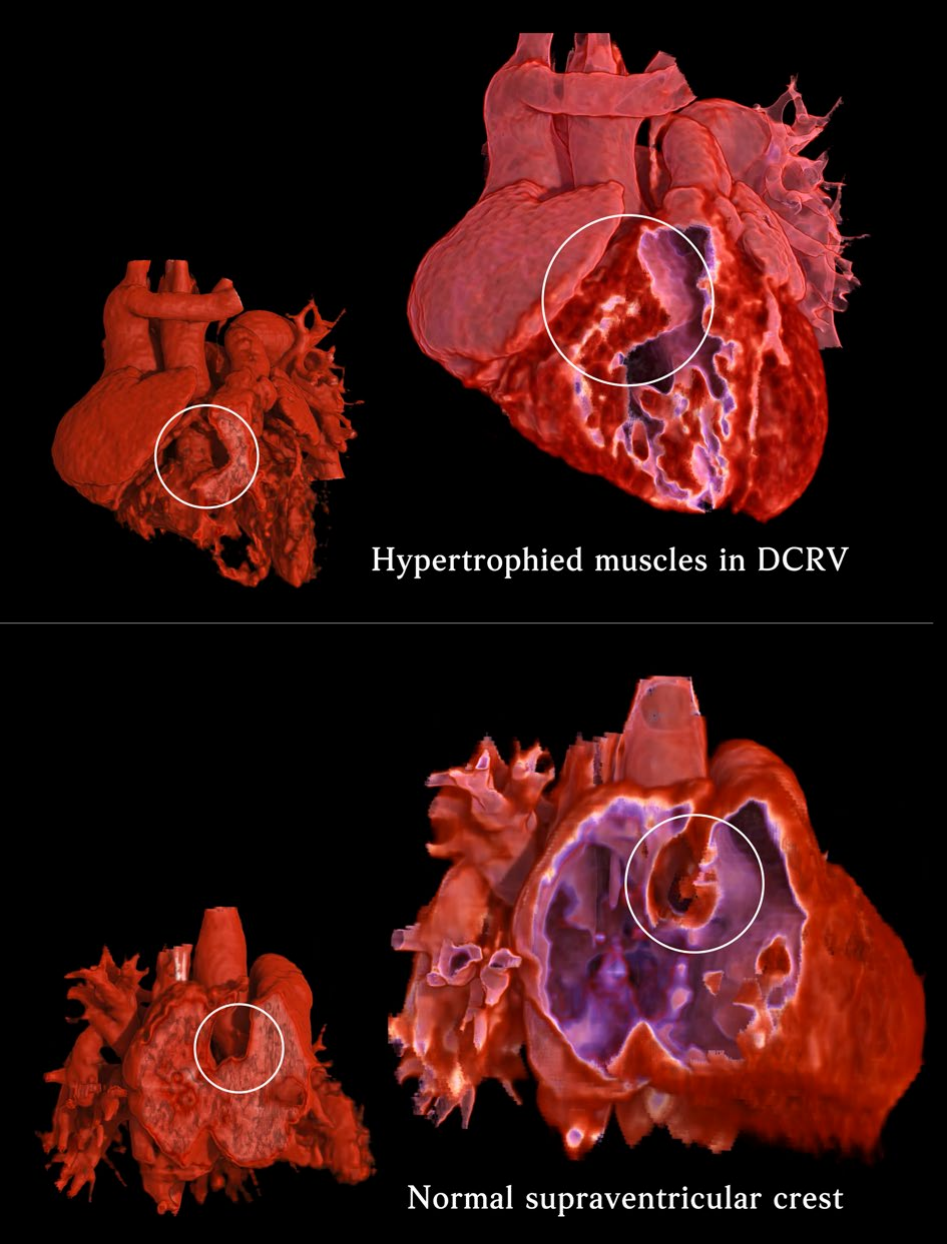
Myocardial models demonstrate muscular thickness and morphology in detail, enabling us to differentiate a normal supraventricular crest (lower row), which is supposed to be just a hollow infolding of the RV wall, from a “solid” hypertrophied muscular ridge in DCRV (upper row). Whereas with regular luminal volume rendering (smaller pictures in the lower left), the models simply outline areas with contrast filling. We see a protrusion into the right ventricle in both normal (lower left in lower row) and DCRV (lower left in upper row) patients, but we are unable to evaluate whether it is caused by solid hypertrophied muscles or just a hollow infolding. All models were frozen at the end-systolic phase.

Since observing cardiac muscles is so crucial in DCRV, we created myocardial models tailored explicitly for the muscular portions of the cardiac chambers during CT post-processing. By rendering the muscles and not the chambers, you get this realist effect and it becomes so easy to spot unusual muscles. (Supplemental video) Our questionnaire results confirmed that myocardial models are effective image addendums that subjectively increase physicians’ confidence. It was also during this process that we discovered most hypertrophied muscular bundles in our DCRV cases (*n* = 21; 91.3%) occur just inferior to the level of the supraventricular crest in RV, not just anywhere randomly. The morphology consistency across different patients is surprisingly high. Maron et. al first proposed the idea back in 1973^24^ that DCRV might result from an acquired process in which the supraventricular crest slowly hypertrophied due to increased turbulent flow and shunting from VSD thus the high co-occurrence of DCRV and VSD. Our data supported the hypothesis in that a markedly high degree of our cases had hypertrophied muscle bundles involving or just inferior to the supraventricular crest. Thus it was only reasonable that we chose to measure uniformly close to the level of the supraventricular crest in RV. Other than that, as our myocardial model was able to directly demonstrate muscular thickness and morphology, it enables us to differentiate a normal supraventricular crest, which is supposed to be just an infolding of the RV wall^25^, from a solid hypertrophied ridge in DCRV. Whereas with usual volume rendering, both show indentations in the luminal area and it wouldn’t be easy to tell if condensed muscles are present or the infolding is simply wider. (Fig. 5).

In recent decades, cardiac magnetic resonance has been gaining popularity for the evaluation of pediatric congenital heart diseases with no radiation exposure^26^. However, cardiac magnetic resonance is often costly and time-consuming in Asia. For pediatric patients, this means heavy sedation or anesthesia in most cases, which is not always feasible due to tight schedules and shortage of anesthesia manpower. CT is often considered more cost-effective and accessible than cardiac magnetic resonance here, requiring only light sedation in the pediatric population at the cost of acceptable radiation exposure^16^. We were thereby able to collect such a considerable amount of CT images in DCRV over the past 10 years. Of course, diagnosis of DCRV can always be made with cardiac catheterization, but it would be more invasive and costly than any of the image modalities^17^.

The major limitation in our study involves patient selection. We were unable to recruit patients with isolated DCRV due to its rareness and therefore our data only applies to the majority that has concomitant simple VSDs. There is also the possibility of selection bias in that our patients might have exhibited more worrying clinical symptoms than average VSD patients to warrant an operation, which explains the high DCRV occurrence rate. The presence of other cardiac lesions may complicate the process of measurements which is also not investigated in this study. The results of this study, however, still prove CT to be a reliable modality in the diagnosis of DCRV under most circumstances.

Image post-processing, especially volume rendering, is a time-consuming process that can only be properly done manually, making it unrealistic to include myocardial models in every examination. Nevertheless, its potential to assist in surgical planning should not be underestimated.

In this study, we wish to raise awareness of DCRV in simple VSD cases among cardiologists, cardiothoracic surgeons, and radiologists. Right ventricular constrictive ratio showed promising diagnostic accuracy for the disease, with a cutoff value of less than 20.1%. We share our observations regarding this disease on CT in hopes of enhancing diagnosis before a patient enters the operating room. Our animated myocardial model brings about great potential in providing pediatric cardiology specialists with a better perception of DCRV morphology.

## Supplementary Information

Supplementary Information 1.

Supplementary Information 2.

## Data Availability

The datasets used and/or analyzed during the current study available from the corresponding author on reasonable request.
