# Supplementary material for: Dynamic cardiac computed tomography characteristics of double-chambered right ventricle
Source: Sci Rep. 2022 Nov 29;12:20607. doi: 10.1038/s41598-022-25230-1 (PMC9708647; doi:10.1038/s41598-022-25230-1)
Supplement: Supplementary file 1 — Supplementary Information 1. [file 41598_2022_25230_MOESM1_ESM.pdf]

**Supplemental GIF Legend**

This is an animated myocardial model we generated for preoperative inspection. Hypertrophied muscular bundles are found near the level of the supraventricular crest. See how the muscles contract and become more evident during end-systolic phase.
