# Supplementary figures and images for: Dynamic cardiac computed tomography characteristics of double-chambered right ventricle
Source: Sci Rep. 2022 Nov 29;12:20607. doi: 10.1038/s41598-022-25230-1 (PMC9708647; doi:10.1038/s41598-022-25230-1)

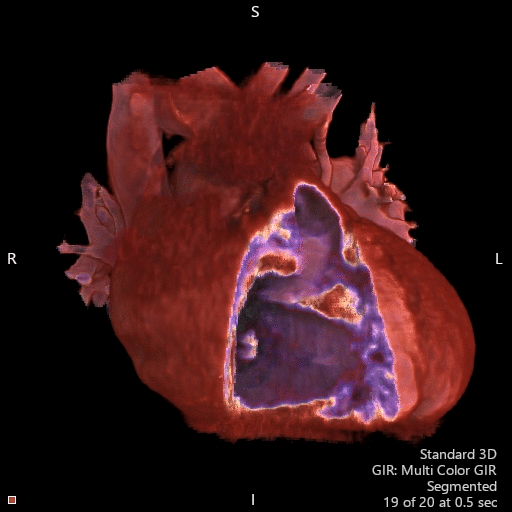

Supplement: Supplementary file 2 — Supplementary Information 2. [file 41598_2022_25230_MOESM2_ESM.gif]
